# Suppressor Analysis Uncovers That MAPs and Microtubule Dynamics Balance with the Cut7/Kinesin-5 Motor for Mitotic Spindle Assembly in *Schizosaccharomyces pombe*

**DOI:** 10.1534/g3.118.200896

**Published:** 2018-11-21

**Authors:** Masashi Yukawa, Yusuke Yamada, Takashi Toda

**Affiliations:** * Hiroshima Research Center for Healthy Aging; † Laboratory of Molecular and Chemical Cell Biology, Department of Molecular Biotechnology, Graduate School of Advanced Sciences of Matter, Hiroshima University, Higashi-Hiroshima 739-8530, Japan

**Keywords:** fission yeast, kinesin, microtubule dynamics, mitotic spindle, suppressor

## Abstract

The Kinesin-5 motor Cut7 in *Schizosaccharomyces pombe* plays essential roles in spindle pole separation, leading to the assembly of bipolar spindle. In many organisms, simultaneous inactivation of Kinesin-14s neutralizes Kinesin-5 deficiency. To uncover the molecular network that counteracts Kinesin-5, we have conducted a genetic screening for suppressors that rescue the *cut7-22* temperature sensitive mutation, and identified 10 loci. Next generation sequencing analysis reveals that causative mutations are mapped in genes encoding α-, β-tubulins and the microtubule plus-end tracking protein Mal3/EB1, in addition to the components of the Pkl1/Kinesin-14 complex. Moreover, the deletion of various genes required for microtubule nucleation/polymerization also suppresses the *cut7* mutant. Intriguingly, Klp2/Kinesin-14 levels on the spindles are significantly increased in *cut7* mutants, whereas these increases are negated by suppressors, which may explain the suppression by these mutations/deletions. Consistent with this notion, mild overproduction of Klp2 in these double mutant cells confers temperature sensitivity. Surprisingly, treatment with a microtubule-destabilizing drug not only suppresses *cut7* temperature sensitivity but also rescues the lethality resulting from the deletion of *cut7*, though a single *klp2* deletion *per se* cannot compensate for the loss of Cut7. We propose that microtubule assembly and/or dynamics antagonize Cut7 functions, and that the orchestration between these two factors is crucial for bipolar spindle assembly.

In eukaryotic cells, the assembly of mitotic spindles is a crucial step in accurate chromosome segregation. The mitotic spindle, a dynamic ensemble of microtubules, microtubule-associated proteins (MAPs) and motor proteins, ensures the proceeding of mitosis by aligning sister chromatids in the equatorial region and segregating them toward opposite poles (Mitchison and Salmon 2001; Woodruff *et al.* 2014). In order to assemble bipolar mitotic spindles, collaborative forces exerted by multiple kinesin motors, collectively called mitotic kinesins, are necessary (Yount *et al.* 2015).

The plus-end directed Kinesin-5 is essential for mitosis and, therefore, influences viability in many eukaryotes from fungi to human beings (budding yeast Cin8 and Kip1, fission yeast Cut7, *Aspergillus* BimC, *Drosophila* Klp61F, *Xenopus* Eg5 and human Kif11) (Enos and Morris 1990; Hagan and Yanagida 1990; Le Guellec *et al.* 1991; Heck *et al.* 1993; Blangy *et al.* 1995). The primary role of Kinesin-5 motors has been shown to be the establishment of spindle bipolarity by driving spindle pole separation. They form homotetramers that crosslink and subsequently slide apart antiparallel microtubules, thereby generating an outward pushing force to separate the two poles (Kashina *et al.* 1996; Kapitein *et al.* 2005). The sole member of the fission yeast Kinesin-5 family, Cut7, is indispensable for mitotic progression. Temperature-sensitive mutations in *cut7* display mitotic arrest with monopolar spindles at the restrictive temperature (Hagan and Yanagida 1990; Hagan and Yanagida 1992). This phenotypic consequence is identical to what is observed in other organisms, including human cells when Kinesin-5 activity is inhibited (Sawin *et al.* 1992; Kapoor *et al.* 2000). Because its function is essential to cell proliferation, Kinesin-5 molecules have been targeted for the development of anticancer drugs (Wacker *et al.* 2012; Ma *et al.* 2014; Dumas *et al.* 2016). However, at present, our knowledge on the physiology and regulation of Kinesin-5 motors is still too limited to draw a complete picture of their functions *in vivo*. It is therefore crucial to obtain a comprehensive understanding of how Kinesin-5 molecules establish spindle bipolarity, and which molecules regulate this kinesin.

Kinesin-5 motors are localized along spindle microtubules during mitosis. However, rather than having a uniform localization pattern on the mitotic spindle, the Kinesin-5 motors are enriched in two regions: at the medial midzone which corresponds to the microtubule plus ends, and near the centrosome which is proximal to the microtubule minus ends (Yount *et al.* 2015). Interestingly, recent work has shown that fungal Kinesin-5 motors are bi-directional: they can move processively toward both plus- and minus-end direction on the microtubules under various conditions (Gerson-Gurwitz *et al.* 2011; Roostalu *et al.* 2011; Edamatsu 2014; Britto *et al.* 2016). It has been reported that individual molecules of budding yeast Cin8 and fission yeast Cut7 initially move toward the minus end, and when concentrated/crowed in clusters on the microtubule, they switch motility from a minus-end- to plus-end-directed manner, thereby generating robust outward force (Britto *et al.* 2016; Shapira *et al.* 2017). This bi-directionality and motility switch may account for the biased localizations of Kinesin-5 on the spindle, though presently, its physiological relevance and significance remain to be established.

Mitotic spindle assembly, *i.e.*, the formation of a microtubule bipolar array, requires another Kinesin family motor, Kinesin-14, which exhibits a minus-end-directed motility (She and Yang 2017). In fission yeast, two Kinesin-14s, Pkl1 and Klp2, counteract Cut7-dependent spindle pole separation (Pidoux *et al.* 1996; Troxell *et al.* 2001; Furuta *et al.* 2008; Braun *et al.* 2009). Pkl1 is localized predominantly to the mitotic spindle pole body (SPB, the fungal equivalent of the animal centrosome), where it forms a ternary complex with Msd1 and Wdr8 (referred to as the MWP complex) (Toya *et al.* 2007; Ikebe *et al.* 2011; Syrovatkina and Tran 2015; Yukawa *et al.* 2015). During early mitosis when duplicated SPBs initially separate, this anchorage generates inward force at the SPB against Cut7-mediated outward force (Yukawa *et al.* 2018). Klp2, on the other hand, is localized to the spindles in a punctate manner and crosslinks microtubule bundles using the N-terminal-located second microtubule binding domain (Braun *et al.* 2009), by which it generates inward forces at the SPBs. Accordingly, the deletion of either *pkl1* or *klp2* suppresses the temperature sensitivity caused by the *cut7* mutations (Pidoux *et al.* 1996; Troxell *et al.* 2001; Rodriguez *et al.* 2008). It is noteworthy that although Pkl1 and Klp2 collaboratively antagonize Cut7, their contributions are not identical; while the *pkl1* deletion (*pkl1*Δ) can completely bypass the requirement of Cut7’s essentiality, *klp2*Δ is incapable of rescuing *cut7*Δ (Yukawa *et al.* 2018).

In this study we sought to identify, in an unbiased manner, genes that antagonize the outward force generated by Cut7/Kinesin-5. To this end, we screened for extragenic suppressors that are capable of rescuing xthe temperature sensitivity of the hypomorphic *cut7* mutants. Consequently, we identified 6 suppressor genes. Subsequent analysis of these genes led us to the proposition that in addition to the MWP complex, microtubule stability and/or dynamics play a crucial role in antagonizing Cut7-mediated outward force. In fact, we have found that chemical perturbation of microtubule stability/dynamics is capable of suppressing the hypomorphic *cut7* mutant phenotype. Remarkably, we have further discovered that treatment with a microtubule destabilizing drug is sufficient to bypass the essential function of Cut7. We discuss how microtubule stability and/or dynamics keep inward and outward forces in balance, thereby assembling bipolar mitotic spindles.

## Materials And Methods

### Strains, media, and genetic methods

Fission yeast strains used in this study are listed in Supplemental Table S1. Media, growth conditions, and manipulations were carried out as previously described (Moreno *et al.* 1991; Bähler *et al.* 1998; Sato *et al.* 2005). For most of the experiments, rich YE5S liquid media and agar plates were used. Wild-type strain (513; Supplemental Table S1), temperature-sensitive *cut7-22*, and *pkl1* deletion strains were provided by P. Nurse (The Francis Crick Institute, London, England, UK), I. Hagan (Cancer Research UK Manchester Institute, University of Manchester, Manchester, England, UK), and R. McIntosh (University of Colorado, Boulder, CO), respectively. Spot tests were performed by spotting 5–10 μl of cells at a concentration of 2 × 10^7^ cells/ml after 10-fold serial dilutions onto rich YE5S plates with or without a drug (thiabendazole, TBZ, T8904, Sigma-Aldrich Co., St. Louis, MO). In some experiments, methyl 2-benzimidazolecarbamate (MBC/CBZ, 378674, Sigma-Aldrich Co., St. Louis, MO) was also used. Some of the YE5S plates also contained Phloxine B (P2759, Sigma-Aldrich Co., St. Louis, MO), a vital dye that accumulates in dead cells and stains the colonies dark pink (Moreno *et al.* 1991). The plates were incubated at various temperatures from 27° to 36° as necessary.

### Preparation and manipulation of nucleic acids

Enzymes were used as recommended by the suppliers (New England Biolabs Inc., Ipswich, MA and Takara Bio Inc., Shiga, Japan).

### Strain construction, gene disruption, and the N-terminal and C-terminal epitope tagging

A PCR-based gene-targeting method (Bähler *et al.* 1998; Sato *et al.* 2005) was used for complete gene disruption and fluorescent-tagging.

### Isolation of revertants from the cut7 mutants

Approximately 2 × 10^7^ *cut7-22* cells/plate were spread on YE5S plates (>20 plates) and directly incubated at 36°. After incubation for several days, revertant colonies were picked up, streaked on YES5 plates and incubated at 36°. Revertants were backcrossed with a *cut7^+^-GFP-kanR* wild-type strain and individual segregants were spread on YE5S plates. If no Ts^-^ segregants appeared, these were assigned as intragenic suppressors, while if Ts^-^ segregants appeared, they were scored as extragenic suppressors. Following this, pair-wise crosses were performed among individual extragenic suppressors. If no Ts^-^ segregants appeared, these two suppressors are classified to belong to the same linkage group. In this way, in total 10 suppressor loci (designated *skf*: *s*uppressor of *k*inesin *f*ive) were assigned. Next, one representative allele from individual *cut7-22 skf* mutants were crossed with a strain containing deletion of *pkl1*, *msd1* or *wdr8*. If Ts^-^ segregants appeared, it is judged that this *skf* gene is not allelic to *pkl1*, *msd1* or *wdr8*. From this analysis, it is concluded that *skf1*, *skf2* and *skf3* are *pkl1*, *wdr8* and *msd1*, respectively, which was confirmed by nucleotide sequencing of these genes in corresponding *skf* mutants.

### Next generation sequencing and annotation of skf genes

At least 5 independent colonies of each *skf* mutant were mixed and cultured in the liquid YE5S. A parental *cut7-22* mutant was also cultured. Genomic DNA was purified according to a standard method (Iida *et al.* 2014). Whole genome sequencing was performed by BGI Next Generation Sequencing (NGS) services (Shenzhen, China). Individual sequence data were analyzed using Mudi (Mutation discovery, http://naoii.nig.ac.jp/mudi_top.html) (Iida *et al.* 2014).

### Fluorescence microscopy and time-lapse live cell imaging

Fluorescence microscopy images were obtained by using the DeltaVision microscope system (DeltaVision Elite; GE Healthcare, Chicago, IL) comprising a wide-field inverted epifluorescence microscope (IX71; Olympus, Tokyo, Japan) and a Plan Apochromat 60×, NA 1.42, oil immersion objective (PLAPON 60×O; Olympus Tokyo, Japan). DeltaVision image acquisition software (softWoRx 6.5.2; GE Healthcare, Chicago, IL) equipped with a charge-coupled device camera (CoolSNAP HQ2; Photometrics, Tucson, AZ) was used. Live cells were imaged in a glass-bottomed culture dish (MatTek Corporation, Ashland, MA) coated with soybean lectin and incubated at 27° for most of the strains and at either 27°, 33° or 36° for the ts mutants. The latter two cultures were incubated in rich YE5S media until mid–log phase at 27° and subsequently shifted to the restrictive temperature of either 33° or 36° before observation. To keep cultures at the proper temperature, a temperature-controlled chamber (Air Therm SMT; World Precision Instruments Inc., Sarasota, FL) was used. The sections of images were compressed into a 2D projection using the DeltaVision maximum intensity algorithm. Deconvolution was applied before the 2D projection. Images were taken as 14–16 sections along the z axis at 0.2-μm intervals; they were then deconvolved and merged into a single projection. Captured images were processed with Photoshop CS6 (version 13.0; Adobe, San Jose, CA).

### Quantification of fluorescent signal intensities

For quantification of GFP-Pkl1, Msd1-GFP, Wdr8-GFP and GFP-Alp4 levels at SPBs in pre-anaphase cells (spindle length < 3.0 μm), a 4 × 4–pixel (0.43-μm square) region of interest (ROI) with maximum sum intensity was determined using the deconvolved and projected images. For quantification of Klp9-GFP located at the spindle midzone during anaphase B (spindle length > 6.0 μm), a 20 × 20–pixel (2.15-μm square) ROI with maximum sum intensity was determined. After subtracting the mean intensity of background signals outside cells, the values of the maximum sum intensities were used for statistical data analysis. For quantification of mCherry-Atb2, GFP-Klp2 and Ase1-GFP located on the metaphase spindle (spindle length < 3.0 μm), maximum sum intensity along individual spindles was determined, and after background subtraction, the values were divided by the spindle length in each cell.

### Statistical data analysis

**Figure 1:**
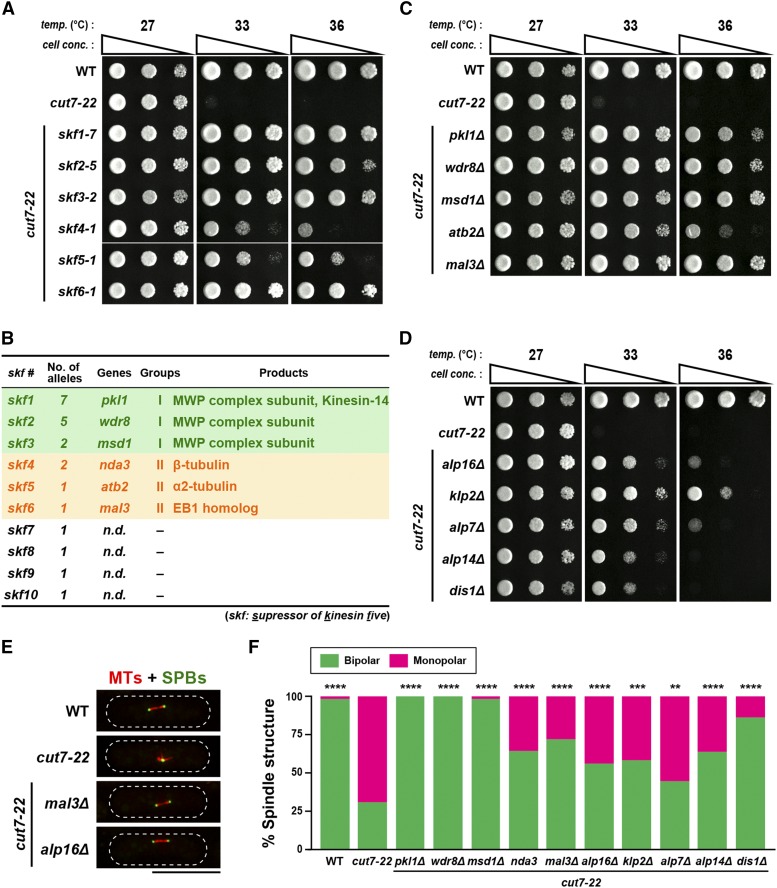
Identification of suppressor genes that rescue the *cut7-22* temperature sensitive mutant. (A) Spot test. Indicated strains were spotted onto rich YE5S agar plates and incubated at 27°, 33° or 36° for 3 d. 10-fold serial dilutions were performed in each spot. *cell conc*., cell concentration, *temp*., temperature. (B) A summary table of *skf* genes. Group I includes genes encoding the MWP complex, while those in Group II encode either tubulins or the microtubule plus-end tracking protein Mal3/EB1. *n.d*.: not determined. (C, D) Spot tests. The same procedures were followed as in (A). (E) Representative images of spindle microtubules in indicated strains. Each strain contains mCherry-Atb2 (MTs) and GFP-Alp4 (SPBs). (F) The percentage of mitotic cells containing bipolar (green) or monopolar spindles (magenta). Individual cells were grown at 33° and spindle morphology was observed. All *p*-values were obtained from the two-tailed χ^2^ test. Data are presented as the means ± SE (≥24 cells). **, *P* < 0.01, ***, *P* < 0.001, ****, *P* < 0.0001. n.s., not significant.

**Figure 2:**
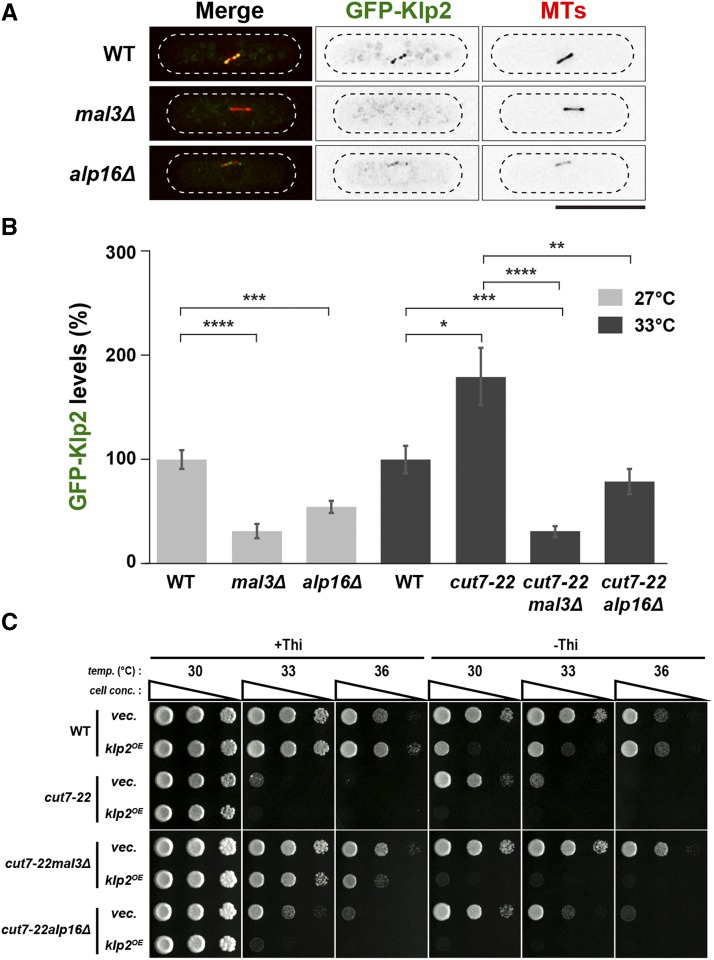
Klp2/Kinesin-14 levels on the spindles determine temperature sensitivity of the *cut7* mutant. (A) Representative images showing mitotic localization of GFP-Klp2 on the spindle microtubule are presented in indicated cells. All strains contain GFP-Klp2 and mCherry-Atb2 (a microtubule marker). Cells were incubated at 27°. The cell peripheries are outlined with dotted lines. Scale bar, 10 μm. (B) Quantification of GFP-Klp2 levels on the spindle microtubule. Individual strains shown in (A) were grown at 27° and a half of each culture was shifted to 33°, while the remaining half was kept at 27°. Fluorescence intensities were measured 2 h later. 33° was used as the restrictive temperature, as fluorescence signals of GFP-Klp2 were quenched rapidly at 36°, which made quantitative measurement of GFP-Klp2 signal intensities difficult. The total values of GFP-Klp2 fluorescence intensities were divided by the spindle length in each cell. The values obtained from wild-type cells incubated at 27° and 33° were set as 100%, and compared to those from other strains incubated at the same temperature. All *p*-values were obtained from the two-tailed unpaired Student’s *t*-test. Data are presented as the means ± SE (≥16 cells). *, *P* < 0.05, **, *P* < 0.01, ***, *P* < 0.001, ****, *P* < 0.0001. (C) Spot test. Individual strains containing a plasmid carrying the *klp2^+^* gene under the thiamine-repressible *nmt1* promoter (Maundrell 1990) or an empty vector were spotted onto minimal plates in the presence (+Thi, 10 μg/ml) or absence (-Thi) of thiamine, and incubated at various temperatures for 3 d. 10-fold serial dilutions were performed in each spot. *cell conc*., cell concentration, *temp*., temperature. Note that temperature sensitivity of *cut7-22mal3*Δ or *cut7-22alp16*Δ became exacerbated upon mild overproduction of *klp2^+^* (33° or 36°). Excessive overproduction of Klp2 (-Thi) was lethal in all the strains at any temperature (Yukawa *et al.* 2018).

**Figure 3:**
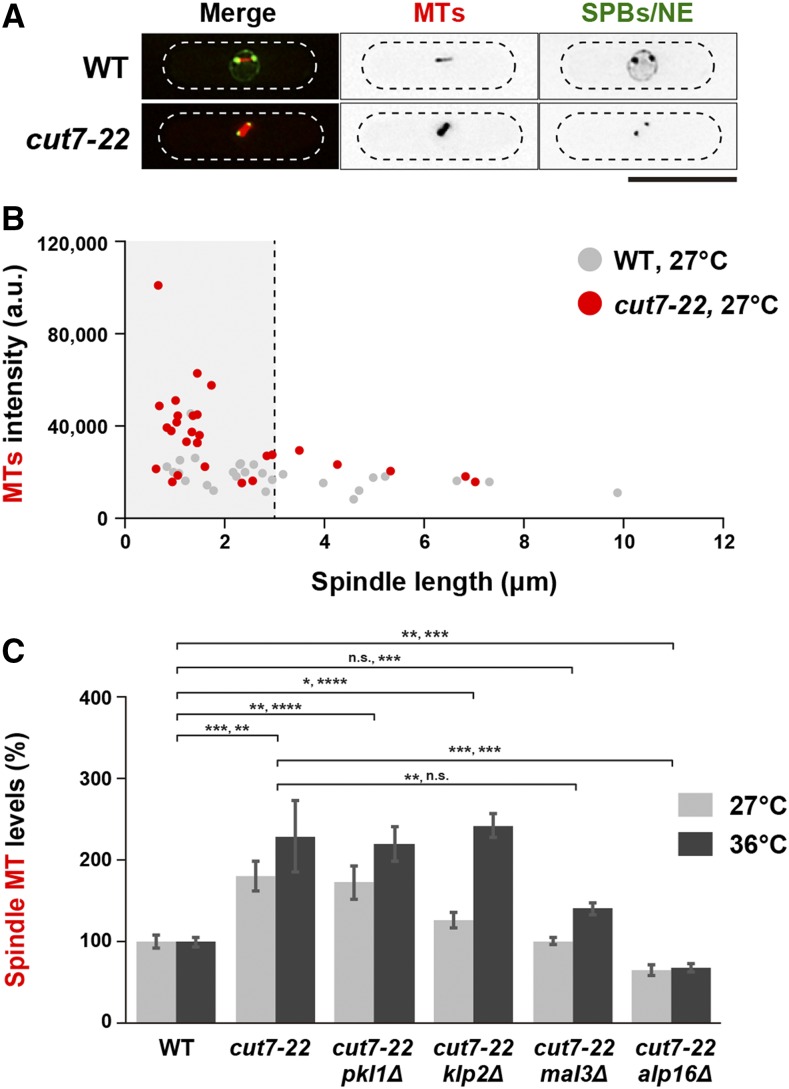
Intensities of the spindle microtubule are increased or conversely reduced in *cut7-22* or mutations that suppress *cut7-22*, respectively. (A) Representative images showing preanaphase spindle microtubules. While wild-type cells contain mCherry-Atb2 (MTs), GFP-Alp4 (SPBs) and Cut11-GFP (a marker for the nuclear membrane and mitotic SPBs) (West *et al.* 1998), *cut7-22* cells contain mCherry-Atb2 and GFP-Alp4. Both strains were incubated at 27° and preanaphase cells (spindle length is < 3 μm) were imaged. Scale bar, 10 μm. (B) Distribution of spindle microtubule intensities in wild-type (gray circles) and *cut7-22* cells (red circles). Spindle microtubule levels of individual mitotic cells shown in (A) were plotted against the spindle length. A vertical dotted line represents the spindle length (3 μm) at metaphase. (C) Quantification of the spindle microtubule. Fluorescence intensities of mCherry-Atb2 were measured in indicated strains that were incubated at either 27° or 36°. Most (>80%) of *cut7-22* mutant cells arrested with monopolar spindles; therefore, spindle intensities of mutant cells displaying short spindles were measured. The values of wild-type cells incubated at 27° or 36° were set as 100%, and compared to those from other strains incubated at 27° or 36°, respectively, for 2 h. All *p*-values were obtained from the two-tailed unpaired Student’s *t*-test. Data are presented as the means ± SE (≥11 cells). *, *P* < 0.05, **, *P* < 0.01, ***, *P* < 0.001, ****, *P* < 0.0001. n.s., not significant.

**Figure 4:**
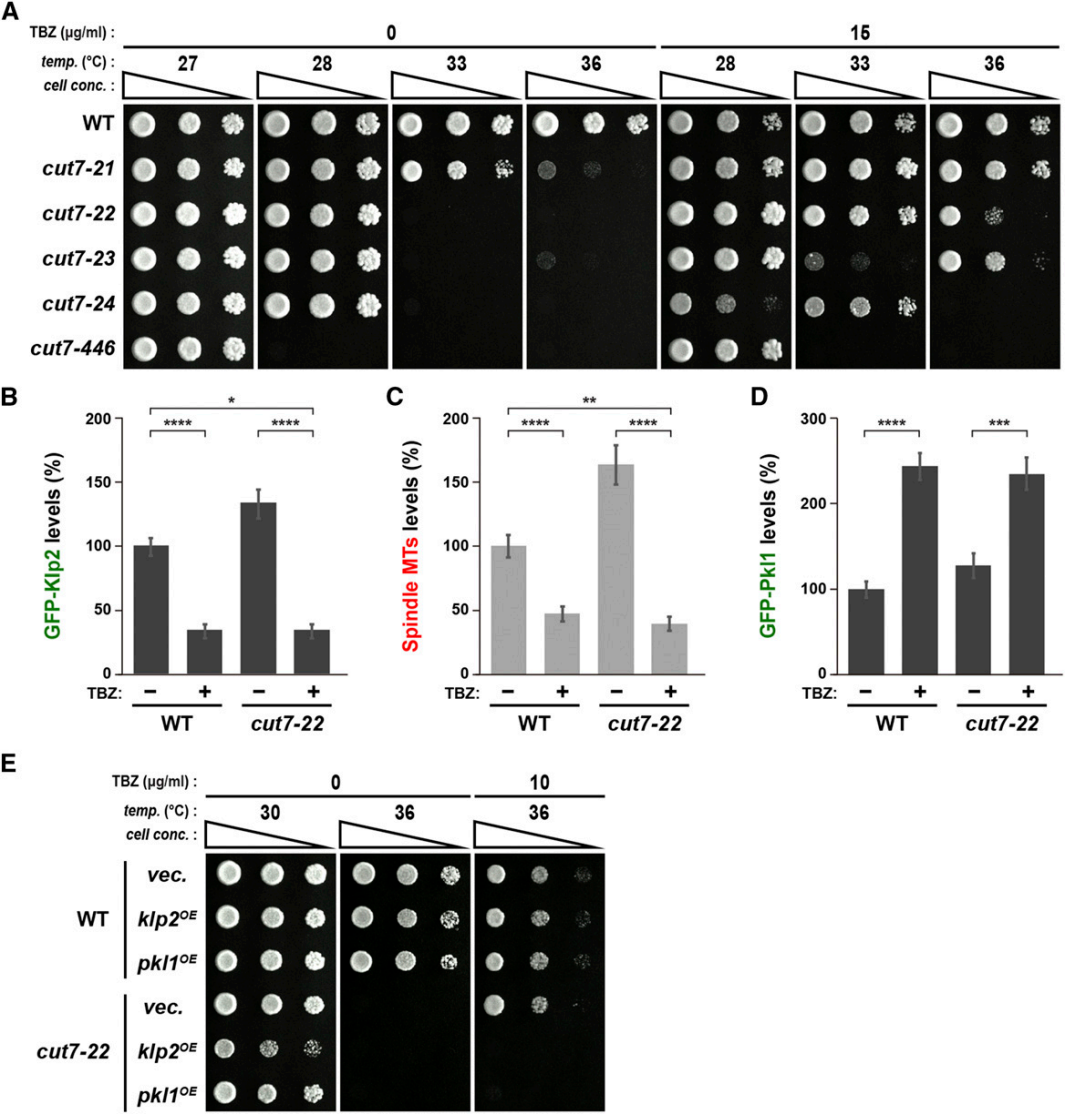
Treatment of a microtubule-destabilizing drug rescues various *cut7* temperature sensitive mutants with reduced levels of Klp2 at the mitotic spindle. (A) Spot test. Indicated strains were spotted onto rich YE5S agar plates in the absence or presence (15 μg/ml) of TBZ, and incubated at 27°, 28°, 33° or 36° for 3 d. 10-fold serial dilutions were performed in each spot. *cell conc*., cell concentration, *temp*., temperature. (B-D) Quantification. Fluorescence intensities of GFP-Klp2 on the spindle microtubule (B), spindle microtubules (C) and GFP-Pkl1 at the mitotic SPB (D) were measured in wild-type and *cut7-22* cells that were incubated at 27° for 12-16 h in the absence or presence of 20 μg/ml TBZ. All *p*-values were obtained from the two-tailed unpaired Student’s *t*-test. Data are presented as the means ± SE (≥10 cells). *, *P* < 0.05, **, *P* < 0.01, ***, *P* < 0.001, ****, *P* < 0.0001. (E) Spot test. Wild-type and *cut7-22* cells were transformed with vector plasmids (*vec*.) or plasmids containing the thiamine-repressive *nmt41-GFP-klp2* (*klp2^OE^*) or *nmt41-GFP-pkl1* (*pkl1^OE^*). Indicated strains were spotted onto minimal plates in the absence or presence (10 μg/ml) of TBZ, and incubated at 30° or 36° for 3 d. 10-fold serial dilutions were performed in each spot. *cell conc*., cell concentration, *temp*., temperature.

We used the two-tailed unpaired Student’s *t*-test to evaluate the significance of differences between the mean fluorescence signal intensities derived from different strains (Figures 2B, 3C and 4B-D and Supplemental Figures S2C-D, S3B-D, S4D, S5 and S6). For testing the significance of differences between frequencies of mitotic cells displaying abnormally extended microtubules in individual strains, we performed the two-tailed χ^2^ test (Figure 1F and Supplemental Figure S8). All the experiments were performed at least twice. Sample numbers used for statistical testing were given in the corresponding figures and/or legends. We used this key for asterisk placeholders to indicate *p*-values in the figures: *e.g.*, ****, *P* < 0.0001.

### Data Availability Statement

Strains and plasmids are available upon request. The authors affirm that all data necessary for confirming the conclusions of the article are present within the article, figures, and tables. Supplemental material available at Figshare: https://doi.org/10.25387/g3.7351985.

## Results

### Isolation of revertants from the cut7-22 temperature sensitive mutants

In order to identify genes counteracting the Cut7/Kinesin-5 function, we isolated spontaneous mutants capable of suppressing the *cut7-22* temperature-sensitive (ts) growth defect at 36°. This screening yielded in total 25 revertants. Subsequent genetic analysis, including backcrossing with a wild-type *cut7*^+^ strain, defined that 3 isolates are intragenic, while the remaining 22 revertants contain extragenic suppressors. Nucleotide sequencing of the *cut7* gene in the 3 intragenic suppressors showed that each revertant contains one additional point mutation within the *cut7 ORF* besides the original mutation (P1021S) (Olmsted *et al.* 2014; Yukawa *et al.* 2018); two mutations were located at the stalk domain of Cut7 (R433Q and L687F), while the third mutation resided at the tail domain (S1027L) (Supplemental Figure S1A).

Pair-wise crosses between each of the 22 strains carrying extragenic suppressors indicated that these mutations consist of 10 linkage groups, designated *skf1-skf10* (*skf*: *s*uppressor of *k*inesin *f*ive). To identify causative mutations within extragenic suppressors, we performed next generation sequencing and analyzed whole-genome sequence data using the Mudi (Mutation discovery), a web tool for identifying mutations based on bioinformatics approaches (Iida *et al.* 2014). This analysis successfully identified individual genes of *skf1-skf6* (Figure 1A and B and Supplemental Figure S1B). These 6 genes could be categorized into two functional groups: Group I (*skf1-skf3*) comprises of genes encoding the components of the MWP complex (Skf1/Pkl1, Skf2/Wdr8 and Skf3/Msd1) as we expected (Yukawa *et al.* 2015), while Group II (*skf4- skf6*) includes those encoding microtubule constituents such as β-tubulin (Skf4/Nda3) (Hiraoka *et al.* 1984), α2-tubulin (Skf5/Atb2) (Toda *et al.* 1984) and the microtubule plus-end tracking protein EB1 homolog (Skf6/Mal3) (Beinhauer *et al.* 1997; Carvalho *et al.* 2003; Asakawa *et al.* 2005). A single *skf4-1/nda3-G56D* strain displayed hyper-sensitivity to a microtubule-destabilizing drug, thiabendazole (TBZ), while an *skf5-1/atb2-G410A* strain showed modest resistance instead (Supplemental Figure S1C). The reason for this difference was currently unknown. The mutation sites within α2- and β-tubulin molecules correspond to the highly conserved amino acids that are located at the outer surface of the α/β-tubulin heterodimer (Supplemental Figure S1D and E). Consistent with these assignments, each deletion of *skf1-skf6* genes suppressed the *cut7-22* ts growth defect at 36° (Figure 1C).

Given that mutations/deletions of genes encoding tubulins or the EB1 homolog suppressed the growth defect caused by *cut7-22*, we next examined whether deletions of other genes involved in microtubule assembly and/or dynamics also display suppressing activity toward *cut7-22*. For this purpose, we examined gene deletions of *alp16* (encoding an ortholog of GCP6, a component of the γ-tubulin complex, γ-TuC) (Fujita *et al.* 2002; Anders *et al.* 2006; Masuda and Toda 2016), *alp7* (encoding an ortholog of the transforming acidic coiled-coil protein TACC) (Sato *et al.* 2004), and *alp14* and *dis1* (encoding XMAP215/TOG microtubule polymerases) (Nabeshima *et al.* 1995; Garcia *et al.* 2001; Al-Bassam *et al.* 2012; Hussmann *et al.* 2016; Matsuo *et al.* 2016), all of which play crucial roles in promoting proper mitotic spindle assembly. Intriguingly, these deletions individually rescued the *cut7-22* ts mutants (Figure 1D). In line with these results, the emergence of monopolar spindles was substantially suppressed in all the double mutants compared to a single *cut7-22* mutant strain (Figure 1E and F). These results indicate that in addition to the impairment of the Pkl1 minus-end directed motor, the perturbation of spindle microtubule assembly including nucleation, polymerization and stabilization, also rescues the *cut7* mutant.

### Klp2/Kinesin-14 levels on the spindles is an important determinant of temperature sensitivity of the cut7 mutant

We previously showed that two Kinesin-14s, Pkl1 and Klp2, generate collaborative inward forces against the outward force exerted by Cut7/Kinesin-5; the deletion of either of these two genes suppresses the *cut7* ts mutants (Yukawa *et al.* 2018). Indeed, Group I suppressors include genes encoding the Pkl1-containing MWP complex (Figure 1B). Hence, one plausible scenario of rescuing the *cut7-22* mutation by Group II suppressors (*skf4-skf6*) would be the downregulation of Kinesin-14 function. To investigate this possibility, we measured the levels of Kinesin-14s, Pkl1 and Klp2, in various strains, at mitotic SPBs and on spindle microtubules, respectively. Interestingly, we found that Klp2 levels were substantially lessened by either *mal3*Δ or *alp16*Δ, whereas conversely, those in the *cut7-22* single mutant were increased (Figure 2A and B).

Intriguingly, in the double mutants (*cut7-22mal3*Δ or *cut7-22alp16*Δ), Klp2 levels were significantly lower than those in *cut7-22* (Figure 2B). The reduction of Klp2 intensities on the spindle microtubules could not be attributed to the gross defects in spindle microtubule structures, as markers for the spindle midzone and antiparallel microtubules (Klp9/Kinesin-6 and Ase1/PRC1, the anaphase B kinesin and the microtubule antiparallel crosslinker, respectively) (Loïodice *et al.* 2005; Yamashita *et al.* 2005; Fu *et al.* 2009) were properly localized to this region in *mal3*Δ or *alp16*Δ cells (Supplemental Figure S2A-D). It is of note that the requirement of Mal3 for the microtubule localization of Klp2 has been previously reported (Mana-Capelli *et al.* 2012), which is in line with our current result. In contrast, Pkl1 levels at mitotic SPBs remained largely unaltered or even became higher in all the strains examined (Supplemental Figure S3A and B). In addition, the levels of the other two components of the MWP complex, Msd1 or Wdr8, were not decreased in *alp16*Δ cells (Supplemental Figure S3C and D).

If the reduced levels of Klp2 on the spindle microtubules were responsible for the suppression of the ts phenotype of *cut7-22* by *mal3*Δ or *alp16*Δ, increased dosage of Klp2 in the double mutants might confer temperature sensitivity again. This was indeed the case; mild overproduction of Klp2 conferred ts growth of *cut7-22mal3*Δ or *cut7-22alp16*Δ double mutants (Figure 2C). These observations imply that the reduced localization of Klp2 at the spindle microtubules, which results from mutations in *mal3* or *alp16*, is the main, if not the sole, reason for suppression of the *cut7* mutants. We envision that in the *cut7* mutants, the increased accumulation of Klp2 on the spindle microtubules contributes to the generation of excessive inward force.

### Intensities of spindle microtubules are increased in the cut7-22 mutant but reduced in the absence of Mal3/EB1 or Alp16/GCP6

In the *cut7-22* mutant, more Klp2 proteins accumulate on the spindles. Moreover, we previously showed that this mutant displays hyper-resistance to TBZ (Yukawa *et al.* 2015). These findings raised the possibility that microtubule numbers are increased, by which spindle microtubules become more stable in the *cut7* mutant than in wild-type cells, leading to Klp2 accumulation and TBZ resistance. To interrogate this point, we directly measured fluorescence signal intensities of mCherry-Atb2 on the spindle microtubule. Intriguingly, the levels of preanaphase spindle microtubules (< 3 μm) were higher in the *cut7* mutant cells than those in wild-type cells at both the permissive and restrictive temperature, 27° and 36° respectively (Figure 3A-C and Supplemental Figure S4A and B).

We further found that the spindle intensities remained higher in the *cut7-22pkl1*Δ or *cut7-22klp2*Δ double mutant, but they were significantly reduced in either of *cut7-22alp16*Δ or *cut7-22mal3*Δ cells (Figure 3C). These results suggest that Klp2 accumulation on the spindles in the *cut7* mutant cells might be attributable to increased spindle levels and that its reduced loading in *cut7-22alp16*Δ or *cut7-22mal3*Δ leads to compromised inward force, resulting in the suppression of temperature sensitivity. It is also noted that the activity of spindle microtubule nucleation/assembly is potentiated when Cut7/Kinesin-5 function is compromised independently of Klp2. However, the levels of Alp4/GCP2, a core component of the γ-TuC (Vardy and Toda 2000) *per se*, were not increased in the *cut7* mutants (Supplemental Figure S4C and D). This result indicates that the potentiation of spindle assembly/nucleation in the *cut7* mutants, if this is the case, does not result from the accelerated recruitment of the microtubule nucleator γ-TuC to the mitotic SPBs.

We further measured the levels of spindle microtubules and Klp2 in Pkl1-overproduced cells. Previous work showed that Pkl1 overproduction recapitulates the phenotype of *cut7* mutants, *e.g.*, the emergence of monopolar spindles, because of the generation of excessive inward force (Pidoux *et al.* 1996; Yukawa *et al.* 2018). We found that the signal intensities of Klp2 were not significantly altered (Supplemental Figure S5A), while those of spindle microtubules were increased as in *cut7-22* cells (Supplemental Figure S5B). Currently, we do not know the uderlying reason for this difference between the *cut7-22* mutation and Pkl1 overproduction with regards to Klp2 levels. It is possible that as Pkl1 is localized to the mitotic spindles as well as the SPBs, Pkl1 on the spindles may structurally interfere with additional recruitment of Klp2 to these locations. In any case, the higher levels of the Klp2 protein on the mitotic spindle appear specifc to the *cut7-22* mutant.

### Drug-induced microtubule destabilization rescues the cut7 temperature sensitive mutation with reduced Klp2 levels at the spindle microtubule

As the temperature sensitivity of *cut7-22* mutants is suppressed by the deletion of various genes that promote microtubule nucleation, assembly and organization, we presumed that simple microtubule destabilization by treatment with anti-microtubule drugs might also effectively rescue the growth defect of the *cut7* ts mutant cells. Therefore, we assessed the impact of TBZ treatment on *cut7* ts mutant strains. As shown in Figure 4A, growth properties of not only *cut7-22* but also all the other *cut7* ts alleles examined were ameliorated on TBZ-containing plates (Figure 4A).

Given that the intensities of spindle-localizing Klp2 and those of the spindles themselves are proportionate and more importantly, that their levels correlate with whether *cut7-22* displays temperature sensitivity (higher) or not (lower) (see Figures 2B and 3C), we reasoned that TBZ treatment would decrease Klp2 levels on mitotic spindles. Indeed, we found that in the liquid culture, the Klp2 levels on the spindles as well as spindle microtubule intensities were reduced (Figure 4B and C). In clear contrast, the Pkl1 levels at the mitotic SPBs were not decreased or even became higher with TBZ treatment (Figure 4D). This was also the case for Msd1 and Wdr8 (Supplemental Figure S6A and B). Intriguingly, mild overproduction of either Klp2 or Pkl1 in *cut7-22* mutants led to the impairment of TBZ-mediated rescuing activity; the ts phenotype of *cut7-22* cells reappeared in the presence of TBZ upon higher dosage of Klp2 or Pkl1 (Figure 4E). This result indicates that microtubule destabilization by either mutations in genes encoding certain MAPs or a chemical reagent could rescue the *cut7* ts phenotype, which is derived from compromised inward force.

### Microtubule destabilization by a drug even bypasses the essentiality of Cut7/Kinesin-5 for viability

**Figure 5:**
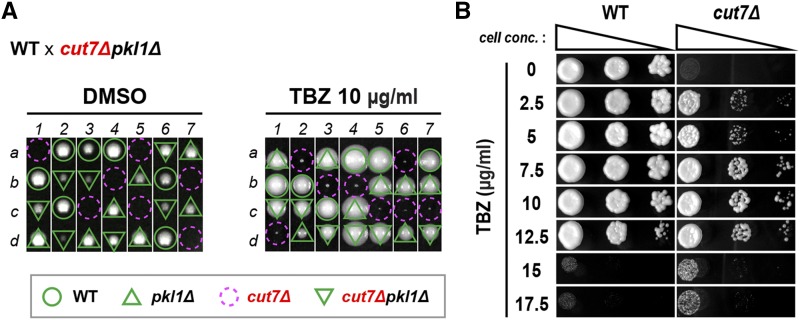
The microtubule-destabilizing drug bypasses the essential function of Cut7/Kinesin-5. (A) Tetrad analysis. Asci formed from diploid cells created by crossing between wild-type and *cut7*∆*pkl1*∆ strains were dissected on rich YE5S plates containing 1% DMSO (left) or 10 μg/ml TBZ (right), and spores were allowed to germinate and proliferate at 27° for 4 d. Representative dissection patterns of individual spores (a–d) in each ascus (1–4) are shown. Assuming 2:2 segregation of individual markers allows the identification of genotype of each segregant. Circles in green indicate wild-type segregants, those in red show *cut7*∆, triangles in green represent *pkl1*∆ and reversed triangles correspond to *cut7*∆*pkl1*∆. Note that all *cut7*∆ spores formed viable, albeit small, colonies in the presence of TBZ, while they were inviable in its absence. (B) Spot test. One of wild-type or *cut7*∆ colonies obtained from tetrad dissection shown in (A) were spotted on YE5S plates in the absence or presence of various concentrations of TBZ, and incubated at 27° for 3 d.

We then performed tetrad dissection of diploids heterozygous for *cut7* and *pkl1* (*cut7^+^/*Δ Δ*/pkl1^+^*), and allowed spores to germinate on plates containing TBZ. Remarkably, this procedure rendered otherwise lethal *cut7*Δ haploid segregants viable; *cut7*Δ cells formed colonies, despite the fact that their size was much smaller than that of wild-type or *cut7*Δ*pkl1*Δ doubly deleted segregants (Figure 5A) (Olmsted *et al.* 2014; Syrovatkina and Tran 2015; Yukawa *et al.* 2017). Continuous cell division of *cut7*Δ cells recovered from TBZ-containing plates upon germination still required the presence of this drug; no colonies were formed on plates in the absence of TBZ (Figure 5B). This indicates that the viability of *cut7*Δ cells is not ascribable to the emergence of suppressor mutations (*e.g.*, *pkl1* or *msd1*) during the period of initial TBZ treatment. We found that very low concentrations of TBZ (2.5 μg/ml), in which no growth defects were apparent in wild-type cells, are sufficient to confer the viability of *cut7*Δ cells. At high concentrations of TBZ (>15 μg/ml), rescuing activity became compromised, as the growth of even wild-type cells was substantially inhibited under these conditions (Figure 5B). The rescue of *cut7*Δ cells was not specific to TBZ, as the addition of a related microtubule-destabilizing compound, methyl 2-benzimidazolecarbamate (MBC), also rendered *cut7*Δ cells viable in a concentration-dependent manner like TBZ (Supplemental Figure S7). Therefore, a moderate disturbance of microtubule stability is crucial to bypass the requirement of Kinesin-5 function. Collectively, the impairment of microtubule stability and/or dynamics renders fission yeast cells viable in the absence of Kinesin-5.

### Gene deletions perturbing spindle microtubule assembly are incapable of bypassing the essentiality of Cut7/Kinesin-5

**Figure 6:**
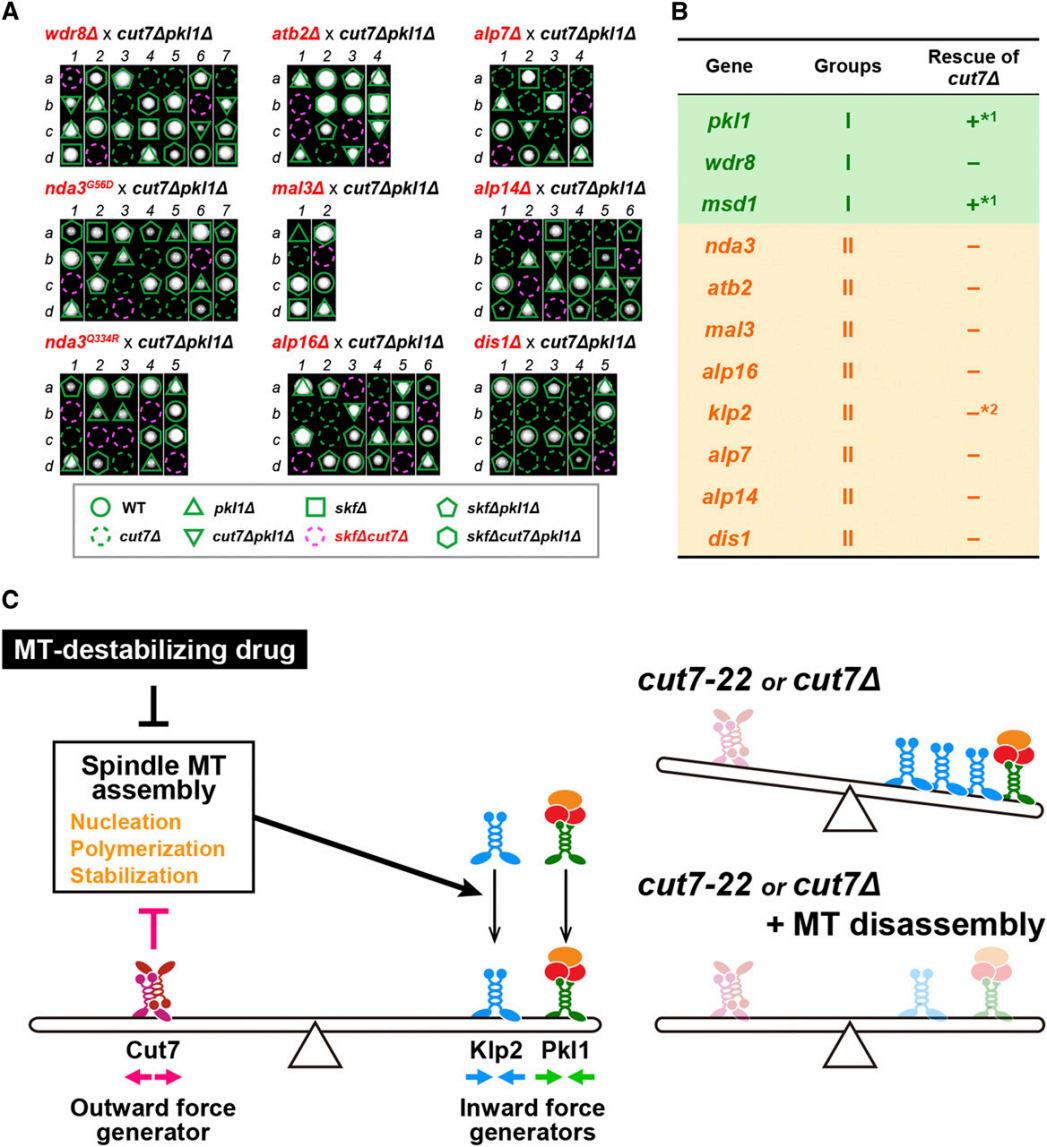
Inability of gene deletions to rescue *cut7*-deleted cells and a schematic model. (A) Tetrad analysis. Tetrad dissections upon crosses between *cut7∆pkl1∆* and mutations or deletions of genes encoding tubulin or various MAPs (collectively shown as *skf* in an outlined frame at the bottom) were performed. Individual spores (a–d) in each ascus (1–4) were dissected on YE5S plates and incubated at 27° for 3 d. Representative tetrad patterns are shown. Assuming 2:2 segregation of individual markers allows the identification of each segregant. Circles in green: wild type; Dotted circles in green: *cut7∆*; Triangles in green: *pkl1∆*; Reversed triangles in green: *cut7∆pkl1∆*; Squares in green: *skf∆*; Dotted circles in red: *skf∆cut7∆*; Pentagons in green: *skf∆pkl1∆*; Hexagons in green: *skf∆cut7∆pkl1∆*. (B) A summary table of suppression profiles toward *cut7∆*. *1: data from (Yukawa *et al.* 2017); *2: data from (Yukawa *et al.* 2018). (C) A model. Bipolar spindle formation requires collaborative balance between mitotic kinesins and microtubule stability/dynamics. Kinesin-5 (Cut7) generates outward force, while Kinesin-14s (Pkl1 and Klp2) generate opposing inward forces. Spindle microtubule stability/dynamics regulated by a cohort of MAPs play a positive role in Klp2 activity, while Cut7 plays an inhibitory role in microtubule nucleation, polymerization and/or stabilization, thereby suppressing Klp2 activity (left). In the *cut7-22* ts mutant, the levels of Klp2 on the mitotic spindles are upregulated. When *cut7-22* or *cut7∆* cells are treated with a microtubule-depolymerizing drug, compromised microtubule dynamics, the reduced level of Klp2 on the spindle and possibly Pkl1 inactivation collaborate to reduce inward forces, by which these cells no longer require Cut7 for survival.

Next, we addressed whether complete deletion of *skf* and other genes involved in microtubule assembly can also bypass the lethality caused by the *cut7* deletion. Tetrad dissection indicated that none of these deletions except for *pkl1* or *msd1* could rescue the lethality of *cut7*Δ cells (Figure 6A and B). As shown earlier (Figure 2), deletions of these genes led to the reduced levels of Klp2 on the spindles. Consistent with this, it is known that unlike *pkl1*, the *klp2* deletion is incapable of rescuing the lethality of *cut7*Δ cells (Yukawa *et al.* 2015). Note that unlike *pkl1* or *msd1*, *wdr8* deletion did not rescue the lethality, but *cut7*Δ*wdr8*Δ*klp2*Δ triple mutants were viable (data not shown). We envisage that even in the absence of Wdr8, cells retain residual Pkl1 activity, thereby rendering *cut7*Δ inviable only in the absence of Klp2. Together, we conclude that the reduction of Klp2 levels on spindle microtubules by TBZ treatment contributes to but is not sufficient for rescuing *cut7*Δ lethality.

As shown earlier, unlike Klp2, the levels of Pkl1 (also Msd1 and Wdr8) at the mitotic SPB were not lessened in either wild-type or *cut7-22* cells upon TBZ treatment (Figure 4D and Supplemental Figure S6). This implies that Pkl1 activity might not be reduced under this condition. To further address the involvement of Pkl1 in TBZ-treated cells, we observed the morphology of mitotic spindles in Pkl1-overproduced *klp2*Δ cells in the presence of TBZ. Interestingly, we found that the emergence of monopolar spindles was substantially suppressed by TBZ treatment (Supplemental Figure S8). This result suggests that inward force generated by Pkl1 is compromised by TBZ treatment in the absence of Klp2 and points toward the possibility that Pkl1 inactivation might in part contribute to the rescue of *cut7*Δ by this drug.

In summary, our genetic analysis indicates that two conditions can rescue hypomorphic *cut7* mutants. The first is the disruption of Pkl1/Kinesin-14 function, which is consistent with previous studies (Pidoux *et al.* 1996; Troxell *et al.* 2001; Olmsted *et al.* 2014; Syrovatkina and Tran 2015; Yukawa *et al.* 2017). The second is the impairment of spindle microtubules by mutations/deletions of genes encoding tubulins and a cohort of MAPs that are important for spindle assembly. We show that the second group lessens Klp2 localization on the mitotic spindle, which could account for the suppression of *cut7* ts mutants (Troxell *et al.* 2001; Yukawa *et al.* 2018). Markedly, continuous treatment by a low dose of the microtubule-destabilizing drug TBZ is capable of not only suppressing the hypomorphic *cut7* mutant but also bypassing the essentiality of Cut7. As even complete deletion of *klp2* fails to rescue the *cut7* deletion (Yukawa *et al.* 2018), TBZ must interfere with some functions other than Klp2 downregulation and Pkl1 inactivation might be involved. We posit that Klp2-independent pathway(s) regulating spindle microtubule stability/dynamics is important to render Cut7 dispensable (Figure 6C).

## Discussion

In this study, we have performed an unbiased genetic screening for suppressors of the *cut7-22* ts mutant, and uncovered a genetic network responsible for conferring lethality to this mutant. We have also found that treatment with the microtubule-depolymerizing drug TBZ rescues the *cut7-22* ts mutant. Remarkably, a low dose of TBZ is capable of even bypassing the essential function of Cut7. Hence, microtubule dynamics and/or assembly kinetics antagonize with Cut7, and the orchestration between these two factors is required for bipolar spindle assembly.

### Compromised microtubule stability/dynamics ameliorate Kinesin-5 deficiency

Previous work in HeLa cells has shown that the perturbation of microtubule stability/dynamics by either knockdown of the chTOG MAP (an ortholog of Alp14 and Dis1) or treatment with low concentrations of microtubule-disruptive drugs (Nocodazole and Vinblastin) can effectively rescue monopolar spindle phenotypes induced by Eg5/Kif11 inactivation (Kollu *et al.* 2009; Florian and Mayer 2011), and a similar observation is also reported in U2OS cells (Kollu *et al.* 2009). These results are wholly consistent with what we observe in fission yeast, indicating evolutionary conservation of Kinesin-5 function. It would, therefore, be worth investigating whether HSET/Kinesin-14 levels on the spindle microtubule are reduced under these conditions, which is observed for fission yeast Klp2/Kinesin-14 under analogous conditions.

### Cut7/Kinesin-5 regulates microtubule stability and/or dynamics

We show that in the *cut7-22* mutant, intensities of spindle microtubules are significantly increased. Furthermore, consistent with these data, *cut7-22* cells display hyper-resistance to microtubule-destabilizing TBZ (Yukawa *et al.* 2015). These results raise the intriguing question as to whether Kinesin-5 molecules *per se* play any role in microtubule stability and/or dynamics. Previous reports indeed show that Kinesin-5 regulates microtubule stability/dynamics as a microtubule depolymerizing factor (budding yeast Cin8 and human Eg5/Kif11) (Gardner *et al.* 2008; Wang *et al.* 2010), conversely as a microtubule stabilizer (budding yeast Kip1) (Fridman *et al.* 2013) or a microtubule polymerase (*Xenopus* Eg5) (Chen and Hancock 2015). In fission yeast, mutations in genes encoding the components of γ-TuC other than Alp16 are also capable of rescuing *cut7-22* (Rodriguez *et al.* 2008), and interestingly, Cut7/Kinesin-5 reportedly interacts with γ-TuC (Olmsted *et al.* 2014). It is, therefore, possible that by physically binding γ-TuC, Cut7 downregulates microtubule nucleating activity, which would explain the increased spindle intensities in the *cut7-22* mutant. As the γ-TuC levels at the mitotic SPBs are unaltered in this mutant, Cut7 might act as a nucleation-inhibitory factor of the γ-TuC, instead of as a spatial regulator. It is noteworthy that it was previously claimed that Cut7 potentiates γ-TuC activity (Olmsted *et al.* 2014); however this notion is somewhat contradictory with genetic results obtained by the same authors (and this study), in which Cut7 and γ-TuC play opposing, rather than collaborative, roles.

Recently, it has become clear that the XMAP215/Dis1/TOG microtubule polymerase family is also a part of a microtubule nucleator; XMAP215 and Alp14 bind γ-TuC directly and indirectly, respectively, thereby promoting the very first step of microtubule assembly (Flor-Parra *et al.* 2018; Thawani *et al.* 2018). It is tempting to speculate that the microtubule nucleator complex includes both positive (the XMAP215/Dis1/TOG family) and negative regulators (Kinesin-5) in addition to its canonical γ-TuC components (Kollman *et al.* 2011; Paz and Luders 2018). Overall, whether and how Cut7 impacts γ-TuC-mediated microtubule nucleation and elongation is an outstanding issue that should be addressed in the future.

### A low dose of the microtubule-destabilizing reagent bypasses the requirement of Kinesin-5

One surprising observation made in this study is the rescue of *cut7*Δ cells by treatment with a low concentration of TBZ or MBC. As *cut7*Δ*klp2*Δ double mutant cells are inviable, the inhibition of Klp2 activity with this drug treatment is not sufficient to fully account for the suppression, though it would make an important contribution. One possibility is the downregulation of Pkl1-mediated inward force. However, the measurement of Pkl1 intensities in TBZ-treated cells does not support this notion (Figure 4D). Nonetheless, it is possible that Pkl1 activity is suppressed by TBZ treatment, which is observed in Pkl1-overproduced *klp2*Δ cells (Supplemental Figure S8). In both animal and fission yeast cells, treatment with low concentrations of microtubule-disruptive drugs interferes with microtubule dynamics and/or increases the concentration of free (unpolymerized) tubulins (Vasquez *et al.* 1997; Mary *et al.* 2015). Thus, compromised microtubule dynamics *per se* likely suppress the generation of lethal inward force in the absence of Kinesin-5, thereby rendering Kinesin-5 dispensable.

### Alp14 and Dis1 microtubule polymerases play both positive and negative roles in kinesin-dependent bipolar spindle assembly

We show here that the deletion of either *alp14* or *dis1* rescues the hypomorphic *cut7* mutant phenotype, indicating that Alp14/Dis1-dependent microtubule polymerization counteracts Cut7-mediated outward force. Curiously and apparently paradoxically, our previous work showed that either of these two MAPs becomes essential for cell viability in the absence of both Cut7 and Pkl1 (*cut7*Δ*pkl1*Δ) (Yukawa *et al.* 2017), showing that Alp14 and Dis1 play a cooperative role with Cut7 in the absence of Kinesin-14. In this latter situation where Cut7 and Alp14/Dis1 (and other MAPs) functionally collaborate (Rincon *et al.* 2017; Yukawa *et al.* 2017), we previously argued that the plus ends of polymerizing microtubules push the respective opposite SPB, thereby generating outward force. This force is sufficient to separate two SPBs in *cut7*Δ*pkl1*Δ cells.

In the hypomorphic *cut7* mutant cells, on the other hand, microtubule polymerization/stabilization mediated by Alp14 or Dis1 is deleterious to these cells. We hypothesize that this adverse impact elicited by Alp14 or Dis1 is mediated by Klp2, which generates antagonistic inward force against Cut7-dependent outward force (Yukawa *et al.* 2018). Taken together, bipolar spindle formation is under the control of a complex network consisting of multiple kinesins and MAPs, and individual molecules display different characters in a context-dependent manner.

### Concluding remarks

Much attention has been focused on mitotic kinesins as druggable targets in cancer therapeutics (Cosenza and Kramer 2016). In particular, inhibitors against Kinesins-5 and -14 are thought to be promising pharmacological reagents, and several small-molecule inhibitors have been clinically evaluated (Huszar *et al.* 2009). One of downsides, however, is the emergence of a cell population that is resistant to drug treatment. Results obtained in this work provide novel insight into the usage of these inhibitors; the combined treatment of Kinesin-5 inhibitors and microtubule stabilizing reagents such as Paclitaxel (Taxol), which by itself is widely used in cancer chemotherapy (Shi *et al.* 2008; Mitchison 2012; Weaver 2014), would be beneficial for the treatment of drug resistant cancers.
